# Crystal structure of 2,3-di­methyl­maleic anhydride: continuous chains of electrostatic attraction

**DOI:** 10.1107/S2056989015013419

**Published:** 2015-07-22

**Authors:** Ren A. Wiscons, Matthias Zeller, Jesse L. C. Rowsell

**Affiliations:** a Department of Chemistry and Biochemistry, Oberlin College, Oberlin, Ohio 44074, USA; b Department of Chemistry, Youngstown State University, Youngstown, Ohio 44555, USA

**Keywords:** crystal structure, maleic anhydride, carbon­yl–carbonyl inter­actions

## Abstract

In the crystal structure of 2,3-di­methyl­maleic anhydride, C_6_H_6_O_3_, the closest non-bonding inter­molecular distances, between the carbonyl C and O atoms of neighboring mol­ecules, were measured as 2.9054 (11) and 3.0509 (11) Å, which are well below the sum of the van der Waals radii for these atoms. These close contacts, as well as packing motifs similar to that of the title compound, were also found in the crystal structure of maleic anhydride itself and other 2,3-disubstituted maleic anhydrides. Computational modeling suggests that this close contact is caused by strong electrostatic inter­actions between the carbonyl C and O atoms.

## Abstract 2

In the structure of 2,3-di­methyl­maleic anhydride, inter­molecular inter­actions are dominated by perpendicular and anti­parallel ^δ+^C⋯^δ−^O carbon­yl⋯carbonyl inter­actions that give rise to a layered structure, and weak inter-sheet C—H⋯O inter­actions between these layers. Carbon­yl–carbonyl inter­actions are persistent across 13 previously reported crystal structures containing a 2,3-disubstituted maleic anhydride moiety.

## Chemical context   

Maleic anhydride and its symmetrically 2,3-disubstituted derivatives are standard reagents found in nearly all chemical stockrooms due to their importance as metal-organic framework post-synthetic modifiers (Wang & Cohen, 2009 ▸), biomolecule denaturation catalysts (Puigserver & Desnuelle, 1975 ▸), synthesis reagents (Moad *et al.*, 2003 ▸), and temperature and pH-reversible co-polymer grafts (Gao *et al.*, 2009 ▸). Although they are seemingly ubiquitous, comparisons of inter­actions in the solid state of maleic anhydride (Lutz, 2001 ▸) and its disubstituted derivatives have not been discussed. Determination of the structure of the title compound, 2,3-di­methyl­maleic anhydride by single-crystal X-ray diffraction was completed and is reported herein. Computational modeling was also used to determine the inter­molecular inter­actions present in the title compound as well as in other 2,3-disubstituted derivatives.
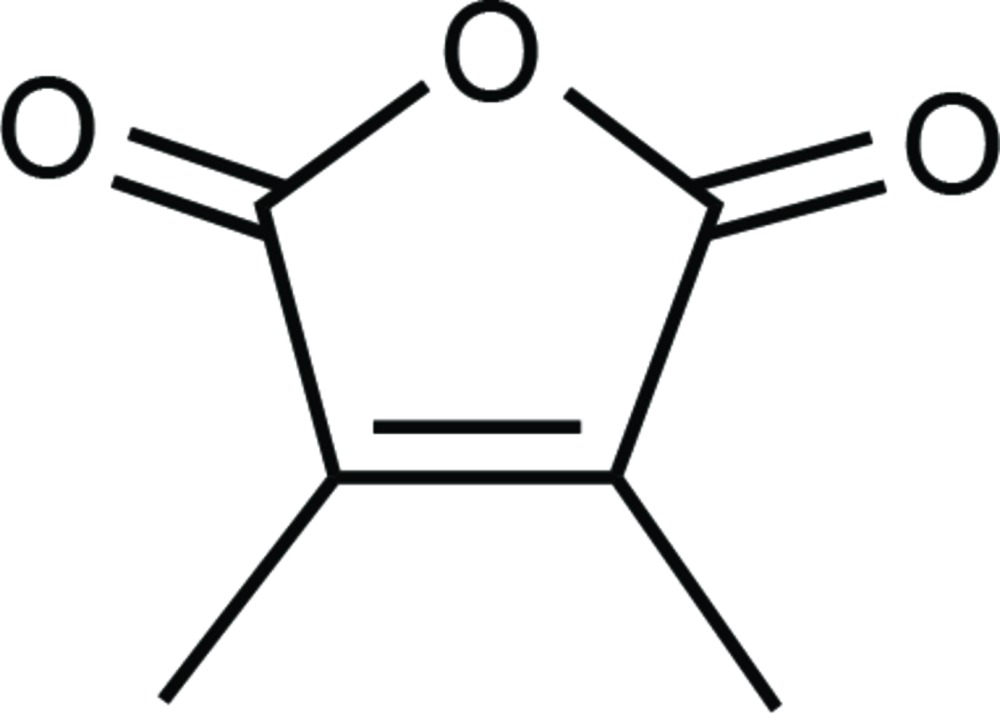


## Structural commentary   

**Figure 1:**
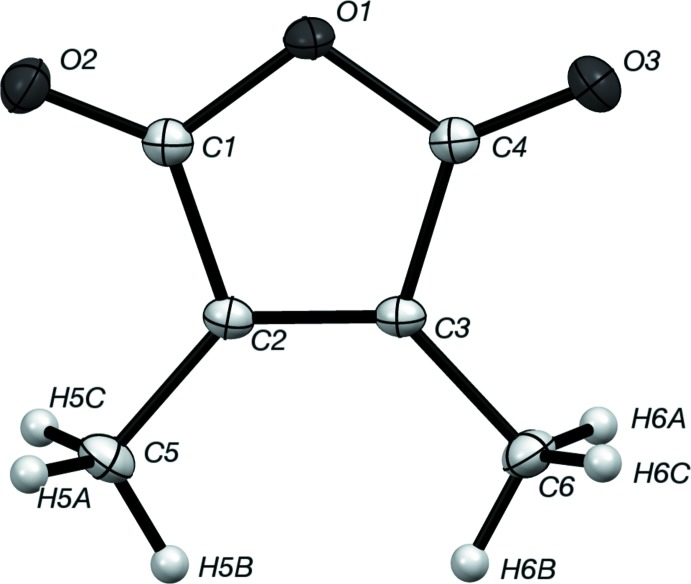
Displacement ellipsoid representation of one mol­ecule of 2,3-di­methyl­maleic anhydride, with non-H atoms drawn at the 50% probability level.

The title compound 2,3-di­methyl­maleic anhydride (Fig. 1 ▸) is a 5-membered cyclic anhydride with a double bond between carbon atoms C2 and C3. The double bond locks the mol­ecule in a planar conformation and stabilizes the acid anhydride against hydration. The lengths of the C—C single bonds between C1 and C2, and C3 and C4 are 1.4841 (11) and 1.4848 (11) Å, respectively, and that of the C=C bond between C2 and C3 is 1.3420 (12) Å, suggesting that the alkene region of the mol­ecule is not delocalized with the carbonyl groups and that the mol­ecule is non-aromatic. The dipole moment of a mol­ecule in the gas phase was calculated as 4.8999 D from DFT B3LYP with a 6-311G(d,p) basis set using *GUUSSIAN03* (Frisch *et al.*, 2004 ▸). All bond lengths and angles are consistent with the mol­ecular structure of unsubstituted maleic anhydride (Lutz, 2001 ▸).

## Supra­molecular features   

**Figure 2:**
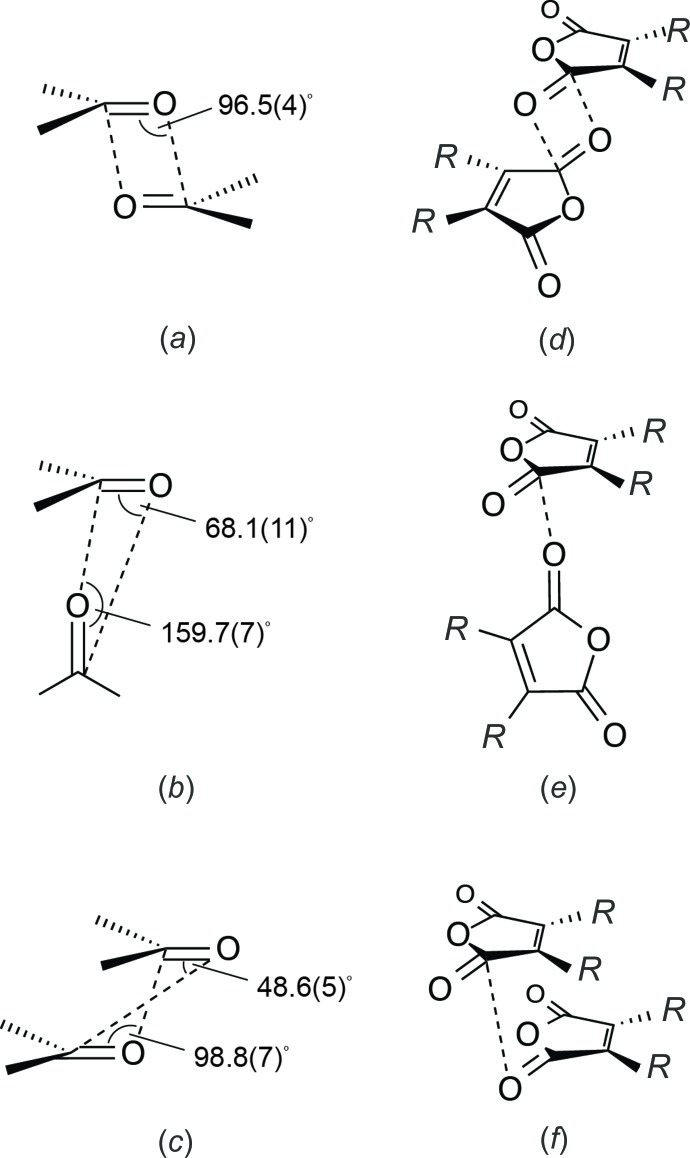
Three carbon­yl–carbonyl inter­action geometries adapted from Allen *et al.* (1998 ▸): (*a*) anti-parallel, (*b*) perpendicular, (*c*) sheared parallel. The three carbon­yl–carbonyl geometries as they apply to substituted maleic anhydrides: (*d*) anti-parallel, (*e*) perpendicular, (*f*) sheared parallel.

In the title compound, close inter­molecular carbon­yl–carbonyl contacts with *d*(^δ+^C⋯^δ−^O) ranging from 2.9054 (11) to 3.0509 (11) Å in length are present, which is well below the sum of the carbon and oxygen van der Waals radii of 3.22 Å (Bondi, 1964 ▸), suggesting a strong attractive inter­action between these two atoms. Close carbon­yl–carbonyl inter­actions, in which *d*(^δ+^C⋯^δ−^O) is < 3.6 Å, persist in 15% of carbonyl-substituted small mol­ecule crystal structures surveyed from the Cambridge Structural Database (CSD) by Allen and colleagues in 1998 (Allen *et al.*, 1998 ▸). Three carbon­yl–carbonyl approach geometries, characterized by specific ranges in angles between the van der Waals radius-overlapped ketonic carbon and oxygen nuclei, were found to describe 71.2% (945 structures) of the observed inter­actions in the 1,328 crystal structures identified as having close carbon­yl–carbonyl contacts: the anti-parallel, perpendicular, and sheared parallel motifs (Fig. 2 ▸). Orthogonality of the inter­acting ketonic nuclei was found to be correlated with multiplicity using *ab initio* calculations to qu­antify inter­action strength (Allen *et al.*, 1998 ▸). Doubly C⋯O connected anti-parallel carbon­yl–carbonyl inter­actions (Fig. 2 ▸ *a*) approached strengths of −22.3 kJ mol^−1^, which is competitive with weak-to-medium-strength classical hydrogen bonds, while singly C⋯O connected perpendicular (Fig. 2 ▸ *b*) and sheared parallel inter­actions (Fig. 2 ▸ *c*) were found to have inter­action strengths reaching −7.6 kJ mol^−1^, which is on a par with strong aromatic stacking inter­actions (Allen *et al.*, 1998 ▸). In addition to the inter­action multiplicity, the anti-parallel geometry is strengthened by π–π inter­actions, lengthening the mean separation distance between carbon­yl–carbonyl contacts relative to those observed in singly connected geometries.

**Figure 3:**
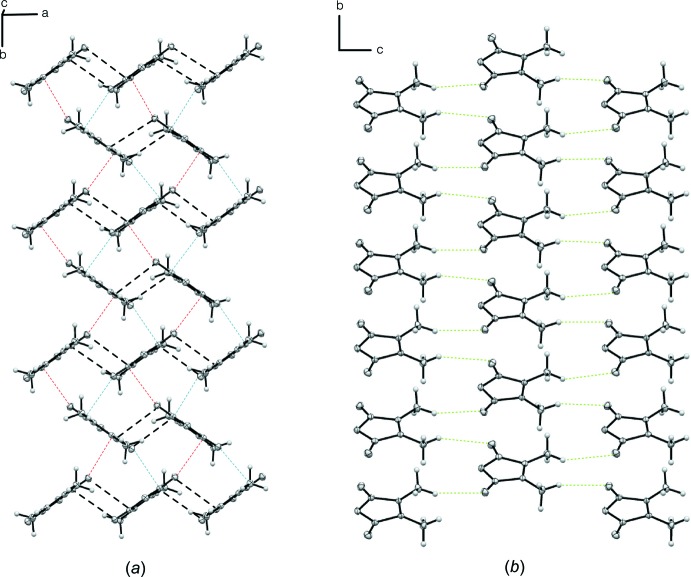
Motifs that arise from non-covalent inter­actions in 2,3-di­methyl­maleic anhydride: (*a*) perpendicular C⋯O inter­actions (red and blue for C1=O2⋯C4^iii^ and C4=O3⋯C2^i^ inter­actions respectively) and anti-parallel carbonyl inter­actions (black, representing C1=O2⋯C4^iv^ and C4=O3⋯C2^v^, respectively), (*b*) weak C—H⋯O inter­actions (green) between sheets (weak C—H⋯O inter­actions within sheets have been omitted for clarity). Symmetry codes: (iii) −*x* + 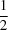, *y* − 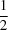, *z*; (iv) *x* + 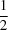, *y*, −*z* + 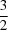; (v) *x* − 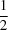, *y*, −*z* + 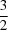. For other codes, see Table 4 ▸.

A survey of thirteen previously determined 2,3-disubstituted maleic anhydride crystal structures demonstrates the persistence of the unsubstituted maleic anhydride’s carbon­yl–carbonyl contacts against steric and electrostatic perturbation (CSD, accessed June 2015; Groom & Allen, 2014 ▸). These 2,3-disubstituted maleic anhydride crystal structures and 2,3-di­methyl­maleic anhydride were characterized in the context of the parameters described by Allen *et al.* (Table 1 ▸ ▸ to 3 ▸). Computational modeling of electrostatic potential and optimized geometric configurations in homomolecular maleic anhydride complexes suggest that non-covalent carbon­yl–carbonyl inter­actions further polarize the inter­acting nuclei, reinforcing the electrostatic attraction, while also polarizing the neighboring anhydride carbonyl, and propagating the carbon­yl–carbonyl inter­actions. The shortest contacts between any two non-H atoms of two mol­ecules are those between the two carbonyl oxygens and the carbonyl C atoms of neighboring mol­ecules. Both anti-parallel and perpendicular motifs are present in the crystal structure (Fig. 3 ▸ *a*). Though the attraction strength for anti-parallel inter­actions is predicted to be greater than that of the perpendicular motif (Allen *et al.*, 1998 ▸), the shortest two contacts belong to the perpendicular inter­actions of O2 and O1 with *d*(^δ+^C⋯^δ−^O) = 2.9054 (11) Å and a C=O⋯C angle of 152.88 (6)° for C1=O2⋯C4^iii^, and *d*(^δ+^C⋯^δ−^O) = 3.0509 (11) Å and a C=O⋯C angle of 143.24 (6)° for C4=O3⋯C2^i^ [symmetry code: (i) −*x*, *y* + 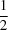, −*z* + 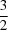, (iii) −*x* + 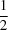, *y* − 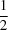, *z*]. The two anti-parallel inter­actions are arranged pairwise between the two carbonyls rather than between carbonyls of the same type (*i.e*., they are not related by inversion symmetry) and they have *d*(^δ+^C⋯^δ−^O) values of 3.220 (11) and 3.259 (11) Å and C=O⋯C angles of 100.86 (5) and 98.89 (5) for C1=O2⋯C4^iv^ and C4=O3⋯C2^v^, respectively [symmetry codes: (iv) *x* + 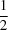, *y*, −*z* + 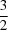; (v) *x* − 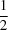, *y*, −*z* + 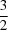]. The greater *d*(^δ+^C⋯^δ−^O) value for the anti-parallel motif relative to the perpendicular motif may be attributed to the π–π inter­actions between the doubly connected carbonyl groups that accompany the ^δ+^C⋯^δ−^O inter­actions.

The perpendicular inter­actions and pairwise anti-parallel inter­actions connect neighboring mol­ecules to form several inter­action motifs (Fig. 3 ▸). Each 2,3-di­methyl­maleic anhydride mol­ecule participates in four perpendicular inter­actions, of which each two are symmetry equivalent: as an electron-density acceptor (through the carbonyl C atom) in two of the four inter­actions and in the other two as an electron-density donor (through the carbonyl O atom). The perpendicular carbon­yl–carbonyl inter­actions associated with both the 2.9054 (11) and 3.0509 (11) Å ^δ+^C⋯^δ−^O distances give rise to pleated chains of 2,3-di­methyl­maleic anhydride mol­ecules that extend parallel to the *b*-axis. There are two parallel chains that arise from the two perpendicular inter­actions with the 2.9054 (11) and 3.0509 (11) Å ^δ+^C⋯^δ−^O separations (C1=O2⋯C4^iii^ and C4=O3⋯C2^i^; Fig. 3 ▸ *a*). The inter­actions join parallel chains and combined they create layers perpendicular to the *c*-axis direction. Mol­ecules within these layers are further connected through the pairwise anti-parallel carbonyl inter­actions and π-stacking, as well as C—H⋯O inter­actions between methyl atom H6*A* and atom O1 (Table 4 ▸). Only weak inter­molecular inter­actions are found between parallel layers of 2,3-di­methyl­maleic anhydride mol­ecules, the most pronounced one being between methyl atom H6*B* and atom O3 (Fig. 3 ▸ *b*).

## Computational modeling   

**Figure 4:**
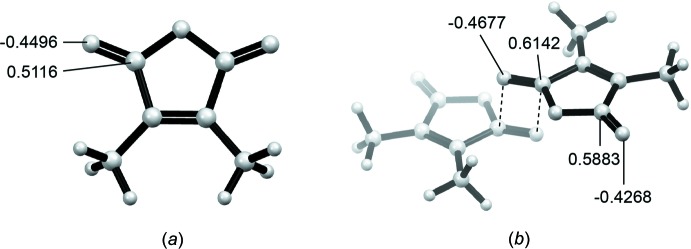
Optimized structures of the single mol­ecule model (*a*) and the 2,3-di­methyl­maleic anhydride dimer with a separation distance of 3.187 Å and (*b*), with indicated Mulliken atomic charges.

To better understand the inter­molecular inter­actions that allow the close contact between the carbonyl C atom and the carbonyl O atom, the anti-parallel carbonyl–carbonyl inter­action between two mol­ecules of 2,3-di­methyl­maleic anhydride was modeled computationally. The perpendicular carbonyl inter­action is not a geometric minimum in the gas phase and thus was not modeled due to the unknown contrib­utions from additional solid-state inter­actions. Geometry optimizations were performed for one mol­ecule of 2,3-di­methyl­maleic anhydride and a dimer of 2,3-di­methyl­maleic anhydride using DFT B3LYP with the 6-31G(d) basis set using *GAUSSIAN03* (Frisch *et al.*, 2004 ▸). Geometry optimization of the two-mol­ecule complex revealed a strong inter­action between the carbonyl O atom and carbonyl C atom with a short *d*(^δ+^C⋯^δ−^O) of 3.178 Å, which is consistent with the value from the crystal structure [3.220 (11) Å] and is below the sum of the van der Waals radii for O and C (3.22 Å). The Mulliken atomic charges of the carbonyl O atom (−0.4496) and carbonyl C atom (+0.5116) suggest that this inter­action is likely electrostatic in nature (Fig. 4 ▸ *a*). Comparison of the computed Mulliken atomic charges of the two-mol­ecule complex with that of a single mol­ecule indicates that both the carbonyl C atom (+0.6142) and carbonyl O atom (−0.4677) atoms participating in the anti-parallel inter­action (Fig. 4 ▸ *b*) are further polarized relative to the free mol­ecule (Fig. 4 ▸ *a*). More inter­estingly, in the two-mol­ecule complex, even the carbonyl C atom not directly involved in the electrostatic attraction is further polarized, with a calculated Mulliken atomic charge of +0.5883 versus +0.5116 in the single-mol­ecule model. These data suggest that the carbon–oxygen electrostatic inter­action on one end of the anhydride draws electron density from the carbonyl C atom on the other and enables 2,3-di­methyl­maleic anhydride to better inter­act with a neighboring carbonyl O atom.

Induced polarization reinforces the overall strength of the carbon­yl–carbonyl network within the crystal structure both between mol­ecules, forming chains through perpendicular inter­actions, and between anti-parallel chains, forming sheets. Based on these calculations, it can be predicted that with increased polarization of the carbonyl carbon and oxygen nuclei, the strength of the inter­molecular inter­action between carbonyls would increase and the shortest contact between the inter­acting nuclei would decrease. Additional inductive effects of dimerization include an increase in the average Mulliken atomic charge of the methyl H atoms (+0.1859) relative to that of the free mol­ecule (+0.1499), which would have the effect of slightly strengthening the weak C—H⋯O attractions that connect layers of mol­ecules associated through the carbon­yl–carbonyl inter­actions.

## Database survey   

The Mulliken atomic charges for thirteen 2,3-disubstituted maleic anhydrides found in the CSD were calculated and their crystal structures analyzed for *d*(^δ+^C⋯^δ−^O) and geometries (Tables 1 ▸, 2 ▸ and 3 ▸). The expected trend is most apparent amongst the set of sheared-parallel carbon­yl–carbonyl inter­actions in which the participating nuclei are isolated from additional non-covalent inter­actions, unlike those found in anti-parallel and perpendicular motifs. This trend supports the prediction that *d*(^δ+^C⋯^δ−^O) decreases with increased carbonyl polarization. The expected trend in anti-parallel *d*(^δ+^C⋯^δ−^O) is disrupted by YUYMIO, a 2,3-di­phenyl­maleic anhydride, whose packing is also guided by edge-face aromatic inter­actions [*d*(C—H⋯centroid] of 3.187 Å). Because of the packing frustration presented by these two competing inter­actions in 2,3-di­phenyl­maleic anhydride, its disruption of the *d*(^δ+^C⋯^δ−^O) trend may be disregarded. These data suggest that C2 and C3 functionalization can affect the carbon­yl–carbonyl inter­action distance for a particular inter­action geometry (anti-parallel, perpendicular, and sheared-parallel) through polarization of the carbonyl group. The persistence of the major inter­actions in maleic acid anhydrides indicates that electrostatic distribution and inter­molecular inter­action-induced polarization of the anhydride’s carbonyls contribute strongly to the mol­ecular packing and are competitive with other common supra­molecular moieties, such as hydrogen-bonding and aromatic stacking.

## Synthesis and crystallization   

Crystals were grown by dissolving 2 g of 2,3-dimethylmaleic anhydride in 100 mL of deionized H_2_O at 373 K. Once dissolved, the solution was slowly cooled to 277 K, crystallizing colorless plates.

## Refinement   

Crystal data, data collection and structure refinement details are summarized in Table 5 ▸. H atoms were positioned geom­etrically and constrained to ride on their parent atoms, with carbon–hydrogen bond distances of 0.95 Å for C—H, and 0.98 Å for CH_3_ moieties, respectively. Methyl H atoms were allowed to rotate but not to tip to best fit the experimental electron density. *U*_iso_(H) values were set to a multiple of *U*_eq_(C) with 1.5 for CH_3_ and 1.2 for C—H.

## Supplementary Material

Crystal structure: contains datablock(s) I. DOI: 10.1107/S2056989015013419/zs2339sup1.cif

Structure factors: contains datablock(s) I. DOI: 10.1107/S2056989015013419/zs2339Isup2.hkl

Supporting information file. DOI: 10.1107/S2056989015013419/zs2339Isup3.cml

CCDC reference: 1412475

Additional supporting information:  crystallographic information; 3D view; checkCIF report

## Figures and Tables

**Table 1:** Anti-parallel interactions (D, , ) in di-substituted maleic anhydrides and the intermolecular carbonyl CO distances in their crystal structures

Molecule	CSD refcode	+ C, C	*d*(CO)	*C*OC
3,4-bis(2,5-dimethylthien-3-yl)furan-2,5-dione	NOYGEN	0.6603, 0.4326	3.063, 3.063	94.98, 94.98
bicyclo(2.2.1)hepta-2,5-diene-2,3-dicarboxylic anhydride	DAJXIV	0.6565, 0.4329	3.088, 3.927	135.40, 85.90
2,3-diphenylmaleic anhydride	YUYMIO	0.6184, 0.4425	3.575, 3.843	75.83, 63.55
4,5,6,7-tetrahydroisobenzofuran-1,3-dione	NADCOL	0.5666, 0.4474	3.108, 3.191	87.26, 83.39
dimethylmaleic anhydride	this work	0.5116, 0.4496	3.220, 3.259	100.86, 98.90
dichloromaleic anhydride	LIZCOM	0.2166, 0.3742	3.211, 3.219	103.01, 102.55

**Table 2:** Perpendicular interactions (D, , ) in di-substituted maleic anhydrides and the intermolecular carbonyl CO distances in their crystal structures

Molecule	CSD refcode	+ C, O	*d*(CO)	*C*OC
bicyclo(2.2.1)hepta-2,5-diene-2,3-dicarboxylic anhydride	DAJXIV	0.6565, 0.4329	3.140, 4.747	137.15, 50.35
3-benzyl-4-phenylfuran-2,5-dione	GUSHOS	0.6410, 0.4266	2.872, 4.257	134.72, 59.61
2,3-diphenylmaleic anhydride	YUYMIO	0.6184, 0.4425	2.913, 3.583	115.98, 80.11
4,5,6,7-tetrahydroisobenzofuran-1,3-dione	NADCOL	0.5666, 0.4474	2.957, 4.360	156.23, 68.51
4,5,6,7-tetrahydroisobenzofuran-1,3-dione	NADCOL	0.5666, 0.4474	3.148, 4.143	124.42, 91.05
dimethylmaleic anhydride	This work	0.5116, 0.4496	2.905, 4.351	152.88, 66.53
dimethylmaleic anhydride	This work	0.5116, 0.4496	3.080, 4.258	130.02, 67.42
4-(4-fluorophenyl)-3-hydroxymaleic anhydride	VEYNIX	0.2632, 0.4579	3.086, 4.271	119.16, 59.94
dichloromaleic anhydride	LIZCOM	0.2166, 0.3742	2.888, 4.346	149.52, 63.38
dichloromaleic anhydride	LIZCOM	0.2166, 0.3742	3.011, 4.275	133.18, 64.81

**Table 3:** Sheared parallel interactions (D, , ) in di-substituted maleic anhydrides and the intermolecular carbonyl CO distances in their crystal structures

Molecule	CSD entry code	+ C, C	*d*(CO)	*C*OC
bicyclo(2.2.2)octa-2,5-diene-2,3-dicarboxylic anhydride	GIQRAZ	0.6408, 0.4410	3.184, 4.092	107.62, 63.77
acenaphthylene-1,2-dicarboxylic acid anhydride	KECPIR	0.6385, 0.4422	3.242, 4.030	102.25, 64.70
2-(1,2-dimethylindol-3-yl)-3-(1-propenyl)maleic anhydride	FARQUL	0.5945, 0.4398	3.243, 4.027	92.18, 55.81
bicyclo(2.2.1)hept-2-ene-2,3-dicarboxylic anhydride	DAJXOB	0.5930, 0.4302	3.434, 3.498	87.57, 81.57
2-phenylmaleic anhydride	ZIVKOE	0.4665, 0.4373	3.847, 4.151	83.27, 97.93

**Table 4:** Hydrogen-bond geometry (, )

*D*H*A*	*D*H	H*A*	*D* *A*	*D*H*A*
C6H6*A*O1^i^	0.98	2.68	3.5004(12)	142
C6H6*B*O3^ii^	0.98	2.69	3.5445(13)	146

Symmetry codes: (i) 
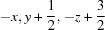
; (ii) 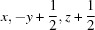.

**Table 5:** Experimental details

Crystal data	
Chemical formula	C_6_H_6_O_3_
*M*_r_	126.11
Crystal system, space group	Orthorhombic, *P* *b* *c* *a*
Temperature (K)	100
*a*, *b*, *c* ()	10.4087(18), 8.5848(15), 13.095(2)
*V* (^3^)	1170.1(3)
*Z*	8
Radiation type	Mo *K*
(mm^1^)	0.12
Crystal size (mm)	0.50 0.31 0.19

Data collection	
Diffractometer	Bruker APEXII CCD
Absorption correction	Multi-scan (*SADABS*; Bruker, 2013 ▸)
*T*_min_, *T*_max_	0.643, 0.746
No. of measured, independent and observed [*I* > 2(*I*)] reflections	16692, 1856, 1688
*R*_int_	0.031
(sin /)_max_ (^1^)	0.735

Refinement	
*R*[*F* ^2^ > 2(*F* ^2^)], *wR*(*F* ^2^), *S*	0.037, 0.101, 1.07
No. of reflections	1856
No. of parameters	84
H-atom treatment	H-atom parameters constrained
_max_, _min_ (e ^3^)	0.53, 0.19

Computer programs: *APEX2* and *SAINT* (Bruker, 2013 ▸), *SHELXS97* (Sheldrick, 2008 ▸), *SHELXL2014* (Sheldrick, 2015 ▸), *SHELXLE* (Hbschle *et al.*, 2011 ▸), *Mercury* (Macrae *et al.*, 2008 ▸) and *publCIF* (Westrip, 2010 ▸).

## supplementary crystallographic information

### Crystal data

**Table:**

C_6_H_6_O_3_	*D*_x_ = 1.432 Mg m^−^^3^
*M**_r_* = 126.11	Mo *K*α radiation, λ = 0.71073 Å
Orthorhombic, *P**b**c**a*	Cell parameters from 4877 reflections
*a* = 10.4087 (18) Å	θ = 3.1–31.3°
*b* = 8.5848 (15) Å	µ = 0.12 mm^−^^1^
*c* = 13.095 (2) Å	*T* = 100 K
*V* = 1170.1 (3) Å^3^	Plate, colourless
*Z* = 8	0.50 × 0.31 × 0.19 mm
*F*(000) = 528	

### Data collection

**Table:**

Bruker APEXII CCD diffractometer	1856 independent reflections
Radiation source: fine focus sealed tube	1688 reflections with *I* > 2σ(*I*)
Graphite monochromator	*R*_int_ = 0.031
ω and phi scans	θ_max_ = 31.5°, θ_min_ = 3.5°
Absorption correction: multi-scan (*SADABS*; Bruker, 2013)	*h* = −15→15
*T*_min_ = 0.643, *T*_max_ = 0.746	*k* = −12→12
16692 measured reflections	*l* = −19→19

### Refinement

**Table:**

Refinement on *F*^2^	Primary atom site location: structure-invariant direct methods
Least-squares matrix: full	Secondary atom site location: difference Fourier map
*R*[*F*^2^ > 2σ(*F*^2^)] = 0.037	Hydrogen site location: inferred from neighbouring sites
*wR*(*F*^2^) = 0.101	H-atom parameters constrained
*S* = 1.07	*w* = 1/[σ^2^(*F*_o_^2^) + (0.0563*P*)^2^ + 0.342*P*] where *P* = (*F*_o_^2^ + 2*F*_c_^2^)/3
1856 reflections	(Δ/σ)_max_ = 0.001
84 parameters	Δρ_max_ = 0.53 e Å^−^^3^
0 restraints	Δρ_min_ = −0.19 e Å^−^^3^

### Special details

**Table:**

Geometry. All e.s.d.'s (except the e.s.d. in the dihedral angle between two l.s. planes) are estimated using the full covariance matrix. The cell e.s.d.'s are taken into account individually in the estimation of e.s.d.'s in distances, angles and torsion angles; correlations between e.s.d.'s in cell parameters are only used when they are defined by crystal symmetry. An approximate (isotropic) treatment of cell e.s.d.'s is used for estimating e.s.d.'s involving l.s. planes.

### Fractional atomic coordinates and isotropic or equivalent isotropic displacement parameters (Å^2^)

**Table:**

	*x*	*y*	*z*	*U*_iso_*/*U*_eq_	
C1	0.21013 (8)	0.04947 (9)	0.74377 (6)	0.01397 (17)	
C2	0.16580 (8)	0.09029 (9)	0.84811 (6)	0.01289 (17)	
C3	0.06175 (8)	0.18143 (9)	0.83852 (6)	0.01265 (17)	
C4	0.03516 (8)	0.20010 (9)	0.72780 (6)	0.01341 (17)	
C5	0.23380 (9)	0.03114 (10)	0.93985 (7)	0.01846 (19)	
H5A	0.2334	−0.0830	0.9392	0.028*	
H5B	0.1902	0.0687	1.0015	0.028*	
H5C	0.3227	0.0687	0.9394	0.028*	
C6	−0.02176 (9)	0.25635 (10)	0.91611 (7)	0.01814 (19)	
H6A	−0.0139	0.3698	0.9108	0.027*	
H6B	0.0048	0.2229	0.9845	0.027*	
H6C	−0.1113	0.2258	0.9043	0.027*	
O1	0.12815 (6)	0.11870 (7)	0.67262 (5)	0.01582 (16)	
O2	0.29960 (6)	−0.02770 (8)	0.71646 (5)	0.01983 (17)	
O3	−0.04983 (6)	0.26794 (8)	0.68539 (5)	0.01860 (16)	

### Atomic displacement parameters (Å^2^)

**Table:**

	*U*^11^	*U*^22^	*U*^33^	*U*^12^	*U*^13^	*U*^23^
C1	0.0154 (4)	0.0128 (3)	0.0137 (4)	−0.0016 (3)	0.0001 (3)	−0.0002 (3)
C2	0.0145 (4)	0.0126 (3)	0.0115 (3)	−0.0023 (3)	−0.0003 (3)	0.0003 (3)
C3	0.0151 (4)	0.0125 (3)	0.0103 (3)	−0.0020 (3)	0.0005 (2)	−0.0004 (2)
C4	0.0149 (4)	0.0125 (3)	0.0129 (3)	−0.0015 (3)	0.0005 (3)	−0.0004 (3)
C5	0.0194 (4)	0.0210 (4)	0.0150 (4)	−0.0005 (3)	−0.0042 (3)	0.0030 (3)
C6	0.0185 (4)	0.0208 (4)	0.0151 (4)	0.0014 (3)	0.0037 (3)	−0.0026 (3)
O1	0.0192 (3)	0.0177 (3)	0.0106 (3)	0.0027 (2)	0.0002 (2)	−0.0011 (2)
O2	0.0188 (3)	0.0195 (3)	0.0212 (3)	0.0035 (2)	0.0035 (2)	−0.0018 (2)
O3	0.0186 (3)	0.0191 (3)	0.0181 (3)	0.0011 (2)	−0.0039 (2)	0.0023 (2)

### Geometric parameters (Å, º)

**Table:**

C1—O2	1.1976 (10)	C4—O1	1.3955 (10)
C1—O1	1.3963 (10)	C5—H5A	0.9800
C1—C2	1.4841 (11)	C5—H5B	0.9800
C2—C3	1.3420 (12)	C5—H5C	0.9800
C2—C5	1.4840 (11)	C6—H6A	0.9800
C3—C6	1.4837 (11)	C6—H6B	0.9800
C3—C4	1.4848 (11)	C6—H6C	0.9800
C4—O3	1.1959 (10)		
			
O2—C1—O1	120.76 (8)	C2—C5—H5B	109.5
O2—C1—C2	130.35 (8)	H5A—C5—H5B	109.5
O1—C1—C2	108.90 (7)	C2—C5—H5C	109.5
C3—C2—C5	131.32 (8)	H5A—C5—H5C	109.5
C3—C2—C1	107.60 (7)	H5B—C5—H5C	109.5
C5—C2—C1	121.08 (7)	C3—C6—H6A	109.5
C2—C3—C6	131.41 (8)	C3—C6—H6B	109.5
C2—C3—C4	107.74 (7)	H6A—C6—H6B	109.5
C6—C3—C4	120.84 (7)	C3—C6—H6C	109.5
O3—C4—O1	121.09 (8)	H6A—C6—H6C	109.5
O3—C4—C3	130.09 (8)	H6B—C6—H6C	109.5
O1—C4—C3	108.80 (7)	C4—O1—C1	106.95 (6)
C2—C5—H5A	109.5		
			
O2—C1—C2—C3	179.19 (8)	C2—C3—C4—O3	177.77 (8)
O1—C1—C2—C3	−0.43 (9)	C6—C3—C4—O3	−1.64 (14)
O2—C1—C2—C5	−1.17 (13)	C2—C3—C4—O1	−0.84 (9)
O1—C1—C2—C5	179.21 (7)	C6—C3—C4—O1	179.75 (7)
C5—C2—C3—C6	0.48 (15)	O3—C4—O1—C1	−178.21 (7)
C1—C2—C3—C6	−179.93 (8)	C3—C4—O1—C1	0.55 (8)
C5—C2—C3—C4	−178.84 (8)	O2—C1—O1—C4	−179.77 (7)
C1—C2—C3—C4	0.75 (9)	C2—C1—O1—C4	−0.10 (8)

### Hydrogen-bond geometry (Å, º)

**Table:**

*D*—H···*A*	*D*—H	H···*A*	*D*···*A*	*D*—H···*A*
C6—H6*A*···O1^i^	0.98	2.68	3.5004 (12)	142
C6—H6*B*···O3^ii^	0.98	2.69	3.5445 (13)	146

Symmetry codes: (i) −*x*, *y*+1/2, −*z*+3/2; (ii) *x*, −*y*+1/2, *z*+1/2.
